# The relationship between mitochondrial health, telomerase activity and longitudinal telomere attrition, considering the role of chronic stress

**DOI:** 10.1038/s41598-024-77279-9

**Published:** 2024-12-30

**Authors:** Mauricio Guillen-Parra, Jue Lin, Aric A. Prather, Owen M. Wolkowitz, Martin Picard, Elissa S. Epel

**Affiliations:** 1 Departamento de Ecología Evolutiva, Instituto de Ecología, Universidad Nacional Autónoma de México, Ciudad de México, México; 2 Posgrado en Ciencias Biológicas, Universidad Nacional Autónoma de México, Ciudad de México, México; 3 Present Address: Department of Psychiatry and Behavioral Sciences and Weill Center for Neurosciences, University of California, San Francisco, CA 94107 USA; 4 Department of Biochemistry and Biophysics, University of California, San Francisco, CA 94107 USA; 5 Division of Behavioral Medicine, Department of Psychiatry, College of Physicians and Surgeons, Columbia University Irving Medical Center, New York, NY USA; 6 H. Houston Merritt Center for Neuromuscular and Mitochondrial Disorders, Columbia Translational Neuroscience Initiative, Department of Neurology, Columbia University Medical Center, New York, NY USA; 7 Robert N Butler Columbia Aging Center, Columbia University Mailman School of Public Health, New York, NY USA; 8 New York State Psychiatric Institute, New York, NY USA

**Keywords:** Mitochondrial health, Telomerase activity, Telomere attrition, Chronic stress, Senescence, Human behaviour, Telomeres, Mitochondria

## Abstract

Telomere attrition is a hallmark of biological aging, contributing to cellular replicative senescence. However, few studies have examined the determinants of telomere attrition in vivo in humans. Mitochondrial Health Index (MHI), a composite marker integrating mitochondrial energy-transformation capacity and content, may be one important mediator of telomere attrition, as it could impact telomerase activity, a direct regulator of telomere maintenance. In this observational longitudinal study, we examined in peripheral blood mononuclear cells (PBMCs), whether MHI predicted changes in telomerase activity over a 9-month period, thus impacting telomere maintenance over this same period of time. We secondarily examined the role of chronic stress, by comparing these relationships in mothers of children with an autism spectrum disorder (caregivers) vs. mothers of a neurotypical child (controls). Here we show that both chronic stress exposure and lower MHI independently predicted decreases in telomerase activity over the subsequent 9 months. Finally, changes in telomere length were directly related with changes in telomerase activity, and indirectly with MHI and chronic stress, as revealed by a path analysis. These results highlight the potential role of chronic stress and MHI as drivers of telomere attrition in human PBMCs, through an impairment of both energy-transformation capacity and telomerase production.

## Introduction

Rate of cell aging is complex and determined by many different measures of cellular function^1^. Telomere attrition is a well understood pathway leading to replicative senescence^2^. Telomeres are the nucleoproteins located at the ends of chromosomes, and are involved in maintaining genomic integrity^3^. Given that nuclear DNA cannot be fully replicated during mitosis (i.e. end replication problem), telomeres shorten with each round of cell division, unless acted upon by different telomere lengthening functions^4,5^. Telomeres generally shorten throughout life in humans and in cells critically shortened, telomeres promote cellular senescence or apoptosis^4,6^. Shorter telomeres are associated with higher risks of developing different diseases and mortality^7,8,9,10^. Thus, telomeres are considered not only as markers of chronological aging, but as markers reflecting biological aging and overall individual condition.

Rate of telomere attrition over years has rarely been studied longitudinally; however, it should reflect pace of replicative senescence, accumulation of telomere damage, for example through exposure to oxidative stress and hence, indicate immune system aging better than cross-sectional measures of telomere length^11^. Interestingly, telomeres can be maintained and even restored through different physiological pathways^12^, such as the regulation of levels of telomerase enzymatic activity^2,13^. Telomerase contains a core RNA component (TERC) and reverse transcriptase (TERT), which together synthesize new telomeric repeats^2,13^, plus some additional non-canonical functions that have been described^14,15,16^. As a result of the telomerase/telomere maintenance processes, there can be great between-individual differences in the rate of attrition/maintenance of telomeres. Thus, it is important to investigate what factors may be involved in the regulation of telomerase activity and, hence, telomere and cellular maintenance.

Telomerase activity in normal immune cells is negatively correlated with age in humans^17^. Further, telomerase activity in normal immune cells has been found to be negatively associated with certain lifestyle factors^18^, such as smoking (^19^, but see^20^), and to poor mental health, such as chronic stress^21,22^. Paradoxically, some studies have reported that high telomerase in peripheral immune cells, particularly in the context of shorter telomere length, is related to indices of chronic adversity and major depression^23,24^. Despite the importance of understanding telomerase regulation, no human studies we are aware of have examined potential upstream biological regulators of telomerase activity in in vivo settings. Furter, few studies in humans have measured telomerase activity, compared to telomere length. Telomerase activity estimation requires a highly unique assay methodology where both enzymatic activity preservation and RNA isolation are required, hence few studies have examined telomerase activity over time. Here, we examined, in vivo, whether a novel index of mitochondrial health has a role in the telomerase/telomere maintenance processes over time, as substantial evidence from in vitro studies suggest that mitochondria can regulate telomere maintenance, as reviewed below.

Mitochondria transform most of the energy required to power all basal cellular functions and the physiological stress response^25^. Several bidirectional biological pathways link mitochondria to telomere stability^26,27,28^. For example, telomere dysfunction can promote impaired mitochondrial respiration and biogenesis, probably through the activation of p53^26,29,30^. In turn, impaired mitochondrial biology can also lead to an increased telomere attrition^27^, for example, through an increased production of reactive oxygen species (ROS), which can ultimately lead to a higher oxidative stress, and in consequence to a higher telomere attrition^31^. Furthermore, it has been reported that mitochondria are constantly interacting with both telomerase components^27^, including TERT, which is proposed to bind to the mitochondrial DNA where it contributes to maintain its stability and function^32,33^. In turn, TERC has been found to be processed inside the mitochondria and then exported back to the cytosol, probably as a molecule giving information to the nucleus about the mitochondrial function^34,35^. Ultimately, it has been found in basic in vitro models that impaired mitochondrial respiration and impaired cellular bioenergetics can cause a faster telomere attrition^36^, possibly through a reduced capacity for telomerase production^37^, as it affects production of other stress mediators^38^.

**Fig. 1:**
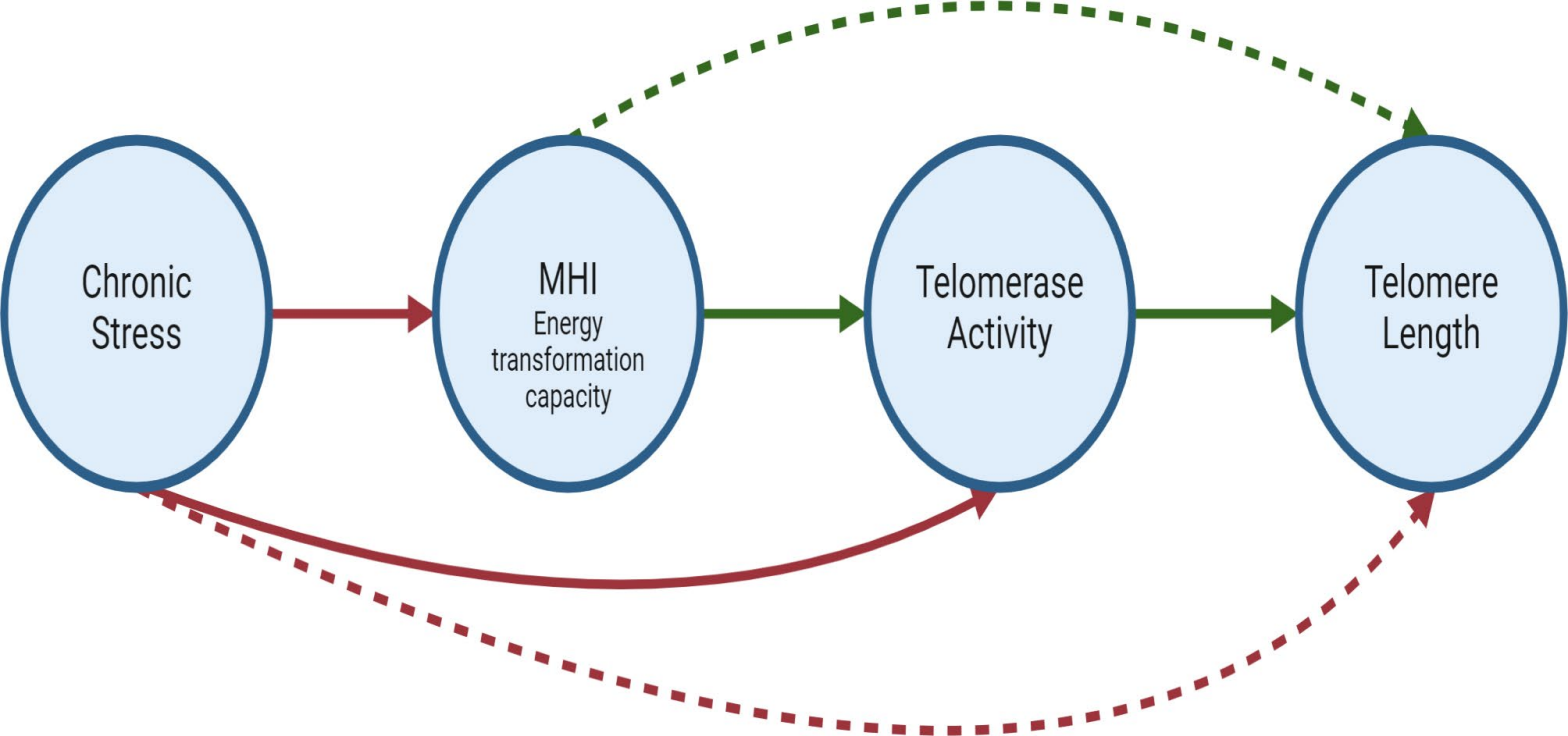
Direct and indirect pathways affecting telomere attrition. Model proposing the pathway regarding how chronic stress and Mitochondrial Health Index (MHI), through direct effects (solid lines) and indirect effects (dotted lines), could be regulating telomerase activity, and in consequence, telomere length. Chronic stress is linked to lower MHI^39^ and telomerase activity^21^ in previous studies. A downregulated MHI could dampen telomerase activity (to be tested here), presumably through an impaired energetic capacity^37^. In consequence, both chronic stress and MHI could be indirectly affecting telomere attrition rate, marked with dotted arrows. Green arrows indicate a positive relationship. Red arrows indicate a negative relationship. Figure created with BioRender.com.

Given the relationships described above, we infer both direct and indirect effects, relative to statistical testing, of chronic stress and mitochondrial-mediated low energetic capacity, over telomere maintenance, as shown in the model of Fig. 1. Importantly, these proposed relationships are only correlational, as no experimental manipulation was performed in any variable to properly test a causation effect.

In this study, we measured in mixed peripheral blood mononuclear cells (PBMCs) the mitochondrial health index (MHI), which integrates measures of mitochondrial respiratory capacity expressed relative to mitochondrial content, producing an index of energy transformation capacity^39^. We test relations between baseline MHI to the changes over time in telomerase enzymatic activity, and to changes in telomere length during a period of 9 months. We determined whether there are direct and indirect roles of MHI in predicting telomere attrition, such as proposed by the model in Fig. 1, by testing if it is a predictor of changes in the enzymatic activity of telomerase and telomere length over the 9-month period. We predicted that individuals with a higher MHI at baseline will evidence a relatively smaller decrease in their telomerase activity over time, compared to individuals with lower baseline MHI. We hypothesized that both MHI and changes in telomerase activity will be associated with changes in telomere length, where individuals with a higher MHI and an increased telomerase activity will have better telomere maintenance over time.

Lastly, we tested if these relationships are moderated by chronic psychological stress (Fig. 1), by comparing mothers of a child with an autism spectrum disorder (i.e. stressed caregivers) with mothers of a neurotypical child (i.e. lower stress control). In this sample, we have previously found that chronic psychological stress is related to worse mitochondrial health^39^. Thus, chronically-stressed mothers with a lower MHI at baseline may have a more rapid decrease in telomerase activity and telomere shortening over a 9-month period.

## Results

### Demographic and baseline biological indices by chronic stress status

The sample of 85 mothers, across both chronic stress status (i.e. caregiver or control), were 43.46 ± 4.96 years old on average, and age did not differ between groups (Table 1). The sample had an average BMI of 25.30 ± 5.00, being also similar between groups (Table 1). Further, BMI did not change on average during the 9-month period (paired t test, t = -0.38, *p* = 0.71).

**Table 1:** Differences between mothers of children with an autism spectrum disorder (caregivers, *n* = 45) and mothers of a neurotypical child (control, *n* = 40) in their demographic characteristics and biological indices at baseline.

	Control	Caregivers	t test	*p* value
**Age**	42.85 ± 4.53	44.11 ± 5.34	1.60	0.11
**Body Mass Index**	24.77 ± 4.43	25.87 ± 5.53	1.38	0.17
**Mitochondrial Health Index**	103.54 ± 31.13	88.97 ± 21.75	-2.47	0.01
**Baseline Telomerase activity**	1.77 ± 0.45	1.83 ± 0.35	1.04	0.30
**Baseline Telomere length**	1.16 ± 0.23	1.22 ± 0.20	-1.76	0.09

The Mitochondrial Health Index (MHI) was different between the two chronic stress groups (Table 1), as previously reported^39^, where caregiver mothers had a lower MHI compared to control mothers (Table 1). Finally, both baseline telomerase activity and telomere length did not differ between high and low chronic stress groups (Table 1).

### Biomarkers by age and BMI

MHI was not related with individuals’ age or BMI (Table 2). Further, telomerase activity at baseline was also not associated with age or BMI (Table 2). Finally, telomere length at baseline was predicted by the individuals’ age (Table 2), as older individuals had shorter telomeres (*β* ± SE = -0.36 ± 0.08, *p* < 0.01), but it was not related with BMI (Table 2).

**Table 2:** Results from linear regression models evaluating the effect of the individuals’ age and BMI over the different biological indices at baseline, and over the change in telomerase activity and telomere length after a 9-month period.

	Mitochondrial Health Index		Baseline Telomerase activity		Baseline Telomere length		Change in Telomerase activity		Change in Telomere length	
	F_1,81_	*p*	F _1,81_	*p*	F_1,77_	*p*	F_1,70_	*p*	F_1,68_	*p*
**Age**	1.21	0.27	1.47	0.23	21.75	< 0.01	1.45	0.23	0.09	0.76
**BMI**	0.06	0.80	0.46	0.50	0.23	0.63	0.11	0.74	0.93	0.34

The change in telomerase activity and the change in telomere length over the 9-month period were not predicted by the individuals’ age or BMI (Table 2).

### Correlations between biological indices at baseline

Telomerase activity at baseline was not significantly related to MHI (F_1,79_ = 2.21, *p* = 0.14) or to chronic stress status (F_1,79_ = 2.39, *p* = 0.12). Further, telomere length at baseline was also not related to MHI (F_1,74_ = 0.31, *p* = 0.58), telomerase activity (F_1,74_ = 0.02, *p* = 0.90) or chronic stress group (F_1,74_ = 0.04, *p* = 0.85).

### Chronic stress, MHI, and changes in telomerase activity and telomere length over time

**Fig. 2:**
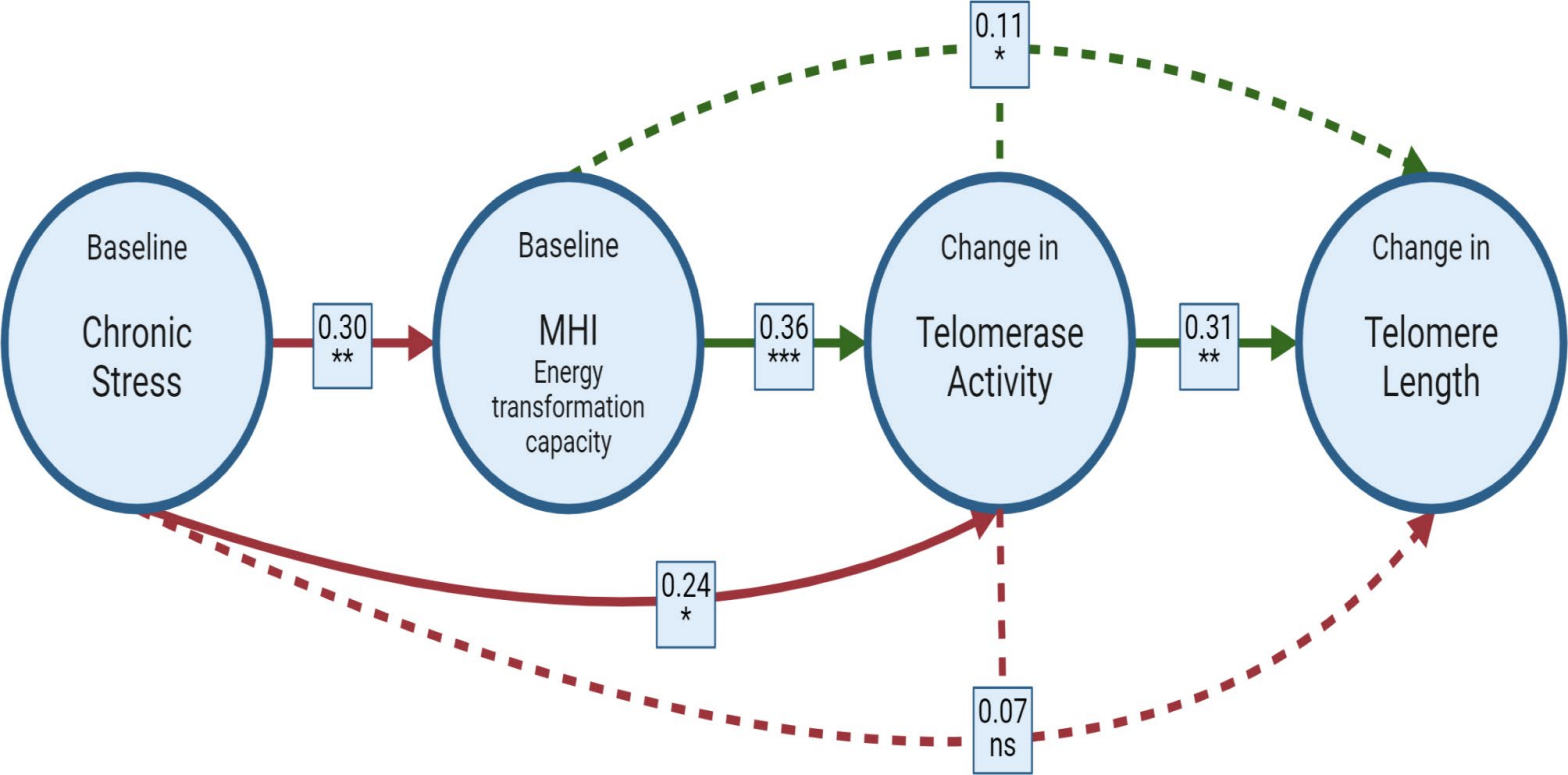
Path model and coefficients for the hypothesized relationships in Fig. 1 leading to telomere attrition. Path analysis testing the direct and indirect effects between chronic stress status (caregiver vs. control mothers), Mitochondrial Health Index (MHI) at baseline, and changes in telomerase activity and telomere length over a 9-month period. Solid arrows indicate direct effects between terms. Dotted arrows indicate indirect effects of chronic stress status and MHI over the change in telomere length, through their effects on another intermediary variable (telomerase activity). In this figure, the numbers indicate the standardized estimated effect of each relationship, and if the effect is significant is noted with asterisks (ns: not significant). Red arrows reflect negative effects. Green arrows reflect positive effects. Figure created with BioRender.com.

The path analysis indicated several direct and indirect effects between our biological indices (Fig. 2).

**Fig. 3:**
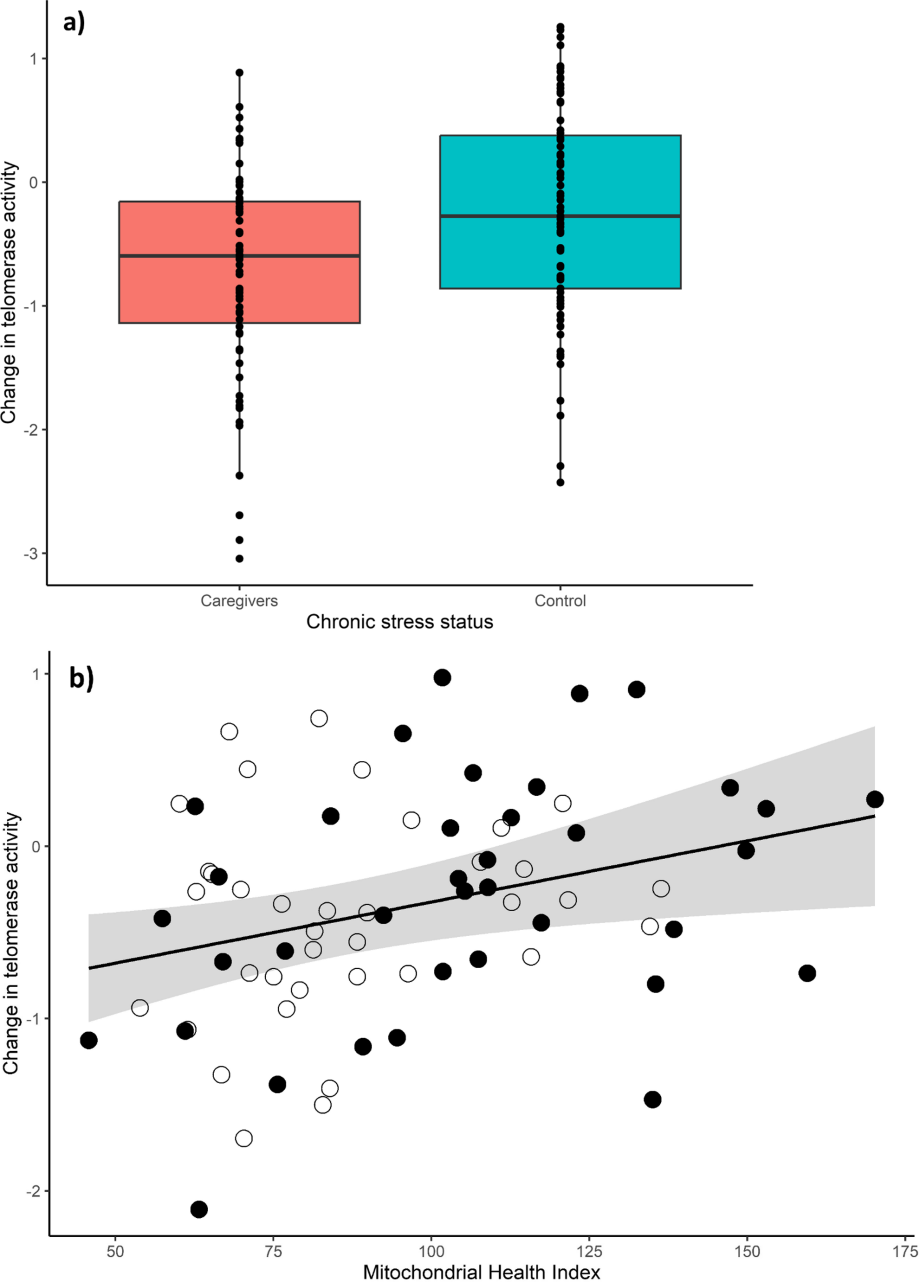
Effects of chronic stress status and Mitochondrial Health Index over the change in telomerase activity after a 9-month period. Changes in (ln) telomerase activity (units/10,000 cells) over a 9-month period explained by the (**a**) chronic stress status (caregivers vs. control mothers), and (**b**) Mitochondrial Health Index (MHI), where open circles are stressed caregiver mothers and closed circles are control mothers, shaded area reflects the confidence interval (95%) for the estimated slope. Positive values indicate an increase in telomerase activity, and negative values indicate a decrease. Model’s r^2^ = 0.24, *p* < 0.001.

Regarding the direct effects, although only correlational, first we see that chronic stress status is associated with MHI, as reported above (*Z* = 2.65, *p* < 0.01). Then, the change in telomerase activity in the 9-month period is negatively related with the chronic stress status (*Z* = 2.24, *p* = 0.02) and positively with the MHI (*Z* = 3.36, *p* < 0.01). Caregiver mothers show a greater decrease in their telomerase activity (-0.73 ± 0.83), than control mothers (-0.25 ± 0.84; Fig. 3a). As predicted, higher baseline values of MHI predict a longitudinal maintenance in telomerase activity, while lower baseline MHI values are associated with a decrease in telomerase activity (Fig. 3b), and this association did not differ by chronic stress group (MHI * Chronic stress F_1,68_ = 0.24, *p* = 0.63).

**Fig. 4:**
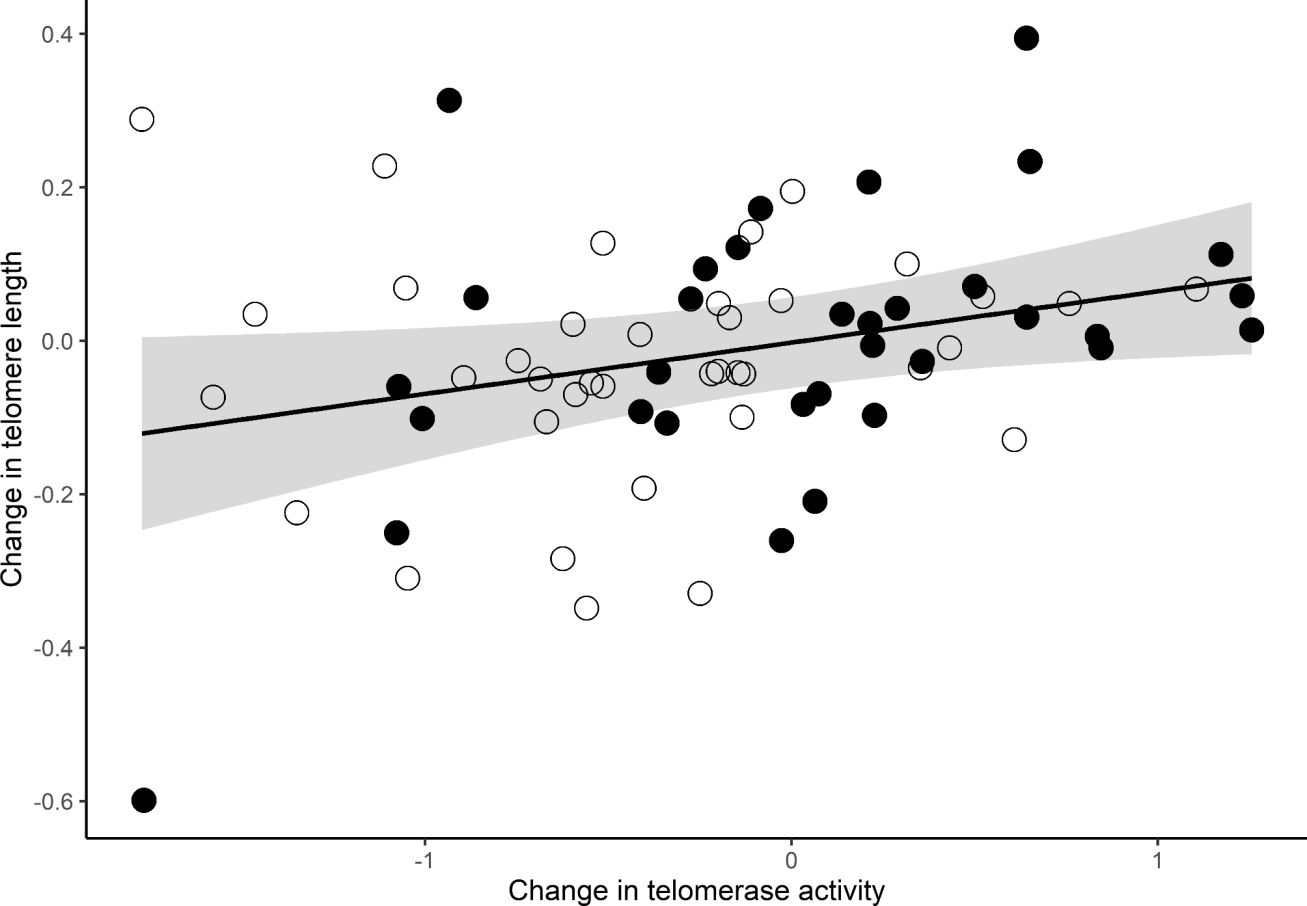
Relationship between the change in telomerase activity and the change in telomere length after a 9-month period. Changes in (ln) telomere length (T/S ratio) over a 9-month period explained by the changes in (ln) telomerase activity (units/10,000 cells) over the same period of time. Positive values indicate an increase in telomerase activity and telomere length, and negative values indicate a decrease. Open circles are stressed caregiver mothers, closed circles are control mothers. Shaded area reflects the confidence interval (95%) for the estimated slope. Model’s r^2^ = 0.10, *p* = 0.05.

The change in telomere length over the 9-month period was not directly predicted by the chronic stress status (*Z* = -0.56, *p* = 0.57) nor by MHI at baseline (*Z* = -0.08, *p* = 0.94). As expected, the change in telomere length was directly related to the change in telomerase activity (*Z* = 2.38, *p* = 0.02). An increase in telomerase activity is associated with a better maintenance of telomere length (Fig. 4). This relationship did not significantly differ between chronic stress groups, although there were marginal differences (Telomerase change * Chronic stress F_1,64_ = 3.45, *p* = 0.07). Control mothers showed a positive relationship between the change in telomerase activity and the change in telomere length (*β* ± SE = 0.39 ± 0.14, *p* < 0.01), as shown in Fig. 4. However, in the stressed caregivers group the slope of the relationship is more flattened (*β* ± SE = 0.03 ± 0.23, *p* = 0.90).

Lastly, the quantification of the indirect effects indicated that, chronic stress status, through its effects on telomerase activity, does not have a statistically significant indirect effect on the change in telomere length (*Z* = 1.63, *p* = 0.10, Fig. 2), although there is the expected trend towards it. Finally, MHI has an indirect effect on the change in telomere length over time, through its effects on the change in telomerase activity (*Z* = 1.94, *p* = 0.05, Fig. 2).

## Discussion

In this study, we found that changes in telomerase activity over time, in human PBMCs, appear partly regulated by mitochondrial health (Mitochondrial Health Index, MHI). Individuals with higher baseline MHI, indicating greater energy transformation capacity, maintained higher active telomerase activity during a 9-month period, compared to individuals with lower MHI, who on average experienced drops in their telomerase activity. Further, the high chronic stress group (caregiving mothers) showed both reduced MHI and a faster decrease in their telomerase activity. Hence, a chronic stressor may amplify the impact of lower energetic capacity on maintaining telomerase activity. Ultimately, having lower MHI, associated with reduced telomerase activity, may speed the rate of telomere attrition, a marker of cellular senescence that has been associated with higher disease and mortality risks, although disease and mortality were not independently assessed in this study. Overall, the predicted relationships of both direct and indirect statistical effects of the model shown in Fig. 1 were supported. These are the first findings we are aware of in humans tracking changes in telomerase activity and telomere length over time, and highlight a novel demonstration of new pathways through which chronic stress and mitochondria may affect telomere dynamics in vivo, described further below.

### Potential mechanisms of mitochondrial health impact on telomerase

Our results suggest that our index of mitochondrial health (MHI) serves as a buffer or positive prognostic factor, as it predicts, in PBMCs, better maintenance of telomerase activity and hence telomere length during a 9-month period. The MHI is a marker reflecting energy transformation capacity per unit of mitochondria content (see Methods), which has been found to be superior in describing mitochondrial “quality” than measures of respiratory capacity or content alone^39^. A higher mitochondrial functional capacity could impact telomerase activity through a higher energy availability. Cells with limited mitochondrial energy transformation capacity (low MHI) may presumably experience greater energetic stress under certain conditions such as immune activation or oxidative stress. Because each cell has a limited energy budget that constrains cellular activities, energy-demanding stress responses can trigger the diversion of energy towards stress processes, and away from longevity-promoting growth, maintenance, and repair (GMR) processes including somatic maintenance and telomerase activity^37^. Such maladaptive energy tradeoff may preferentially affect cells with lower MHI, thus conferring the apparent vulnerability to telomere attrition observed in our study.

Alternatively, higher MHI could also reduce or stop the exportation of telomerase components TERT and TERC from the nucleus, as they are imported into the mitochondria especially during times of high ROS production and oxidative stress, in order to buffer against mitochondrial stress^32,33^. This could allow for a higher telomerase activity in the nucleus, which can ultimately impact on telomere maintenance. However, telomerase components’ non-canonical functions in the nucleus and the mitochondria are not fully understood, and may be independent of one another and from the overall enzymatic activity of telomerase^15,35^. Our results raise the possibility that mitochondria may regulate telomerase activity dynamics over time, but whether this happens due to energetic trade-offs or through another pathway, such as TERT and TERC importation dynamics, remains to be tested in the future. Finally, it could be possible that the apparent regulation of telomerase activity through MHI may also affect the different non-canonical functions of the telomerase^14,15,16^, however this was not assessed in this study.

### What does stress have to do with it?

- *Chronic stress affects mitochondria activity.*

We previously reported that mothers caregiving for a child with an autism spectrum disorder have a lower MHI^39^. MHI can be affected by the emotions and mood of the individuals, even from the hours and days before sampling^39^. Supporting this notion, it has been reported that having a longitudinal higher well-being, evaluated through different psychosocial factors, is related to a higher abundance of mitochondrial OxPhos in postmortem individuals’ brain^40^. Furthermore, mitochondrial respiration and content in PBMCs have been found to be altered in women that have experienced early life stress, reflecting allostatic load^41^. Thus, living with chronic social stress could chronically impair mitochondrial functioning. In male rodents, experimental manipulations have demonstrated that chronic stress can have negative effects on the mitochondrial capacity of individuals^42^. Finally, in humans, at the cellular level, it has been reported that chronic stress can affect both mitochondrial functioning and content, leading to a cellular hypermetabolic state, associated with an increased cellular energy expenditure^36^.

b) *Chronic stress suppresses telomerase activity.*

Chronic stress plays an increasingly recognized important role in cellular aging^43,44^ Individuals experiencing a chronically stressful life situation, such as caregiving stress, have been found to have shorter telomeres^45,46^. Furthermore, basal telomerase activity is another trait possibly affected by chronic stress^47,48,49^, although only a handful of studies have evaluated this relationship in humans. In women, caregivers of either children with autism or dementia patients were associated with lower telomerase activity than control non-stressed women^21,50^. However, opposite results have also been reported, as men and women caregiving for Alzheimer patients show higher telomerase activity compared to control people^51^. Further, in a sample of healthy women, individuals reporting higher perceived chronic stress showed lower telomerase activity^52^. Finally, in older men, high telomerase activity is reported in those having experienced a reduced social support, lower optimism and higher hostility and early life adversity^53^. Regarding mental disorders and their relationship with telomerase activity, although mixed results have been reported^47^, individuals with major depressive disorder seem to consistently show increased peripheral telomerase activity, particularly in men^24,47,54,55^. The present study extends the existing findings by showing that longitudinally, chronic stress, at least in the studied population, differs than depression, and leads to dampened telomerase activity over time, underlying a faster rate of cellular aging.

In this study, we found that the effects of chronic stress on telomerase activity could be partly mediated by the lower mitochondrial health, and subsequent energetic stress, that is found in mothers caregiving for a child with an autism spectrum disorder. Additionally, chronic stress was directly related with the change in telomerase activity over time, independently of their relationship with MHI. Chronic stress can affect telomerase activity through different non-exclusive pathways. For example, individuals under a chronic stress condition are reported to have an altered cortisol secretion^56,57^ and an increased oxidative stress^58^. In turn, chronic oxidative stress is reported to reduce telomerase activity^59^, while glucocorticoids are found to regulate telomerase activity in different directions^60,61^. Thus, the direct relationship of chronic stress over telomerase activity dynamics found here, is likely mediated by other pathways not considered in this paper.

### Mitochondrial health indirectly predicts telomere length attrition

Telomere length and its rate of attrition are markers associated with cellular senescence, reflecting biological aging, since they can predict the individuals’ risk of developing different diseases and all-cause mortality^7,8,9,10^. Hence, it is of great relevance to study how telomerase, a main precursor of telomere maintenance and lengthening, can be regulated over time, as this could directly affect telomere attrition. In this study we found that telomere dynamics can be indirectly altered by both chronic stress and mitochondrial health, as both of them are related to telomerase activity dynamics. In line with our results, in a sample of healthy adults it was found that childhood adversity and lifetime psychopathology were related to an increased mitochondrial DNA copy number and to shorter telomeres^62^. This supports the notion that chronic stress may impair both mitochondrial biology and ultimately telomere maintenance, probably through its effects on telomerase activity, as our results suggest. Interestingly, chronic stress is generally accompanied by a greater perceived stress, while mitochondrial health can be affected by the emotions and mood experienced^39^. Therefore, it would be important to evaluate if different interventions, aiming to reduce stress, could impact on individuals’ mitochondrial biology and telomerase activity, as these seem to improve telomere maintenance^63^, yet this idea was not evaluated in this paper.

Overall, our results in PBMC’s align with recent experimental findings linking impaired mitochondrial respiratory capacity to accelerated telomere shortening rates in cultured human cells^64^. In primary human fibroblasts aged over a 9-month period in vitro, inhibiting mitochondrial respiration accelerated telomere shortening rate by a 7.69-fold^65^. This finding was replicated in cells of individuals with a genetically-defined mitochondrial defect (SURF1 mutation), in which telomere attrition rate was on average 1.62-fold faster than in control cells with normally functioning mitochondria^65^. Moreover, on both models, cells with low mitochondrial health also exhibited accelerated epigenetic aging, based on epigenetic clocks trained to predict age in human tissues^65^. This suggest that the accelerated telomere shortening induced by mitochondrial defects indeed reflects accelerated biological aging, supporting the results of the present study.

Limitations. Finally, it is worth noting that our measures of mitochondrial health, telomerase activity and telomere length were all calculated in peripheral blood mononuclear cells (PBMCs). There is evidence indicating differences in telomerase activity and telomere length between the different immune cell types^66^, where B cells seem to have higher telomerase activity and longer telomeres than T cells^67^. Additionally, mitochondrial function can also differ between B and T cells, and in T cells, activated and inactivated cells may differ in both mitochondrial activity and content^68^. Thus, our results showed here could be partly influenced by differences in cell composition between individuals, and within individuals after the 9-month period. Further, MHI was related with the time the samples spent in the freezer (see Methods). However, we cannot fully rule out the potential confound of freezer time statistically, as it is also confounded with the chronic stress, since samples from caregivers were mostly collected before samples from control mothers. Differences in storage time could indeed affect levels of mitochondrial enzymatic activity. However, when running separate analyses for caregivers and controls, MHI is still related to psychological distress, even after controlling for storage time differences between individuals^39^, suggesting that differences in MHI are indeed linked to the chronic stress of the individuals^39^. Further studies are needed to replicate the chronic stress/mitochondria effect. Another limitation is that we only measured MHI at one timepoint. We therefore do not know how stable it was over time for individuals in our study. We know from work in progress that PBMCs MHI has high stability within the day, and we observe changes on average from 4 to 6% from morning to evening (^69^ in progress). Further, in a study of one subject sampled over time, MHI showed some stability but also changed over weeks^70^. Despite the possible change over time in MHI within individuals, which would tend to decrease/underestimate true effect sizes, we nevertheless found that the individuals’ baseline MHI still predicted their changes in telomerase activity and telomere length. Lastly, telomerase activity in brain may be regulated differently from that in peripheral blood cells^71^, so the present findings should not be uncritically extrapolated beyond PBMCs.

## Conclusion

In summary, our data in PBMCs from healthy midlife women show that chronic stress and low mitochondrial health are associated with a more pronounced reduction of telomerase activity over a 9-month period. These effects consequently contribute to an accelerated rate of telomere attrition, shown by the association between a decrease in telomerase activity and accelerated telomere attrition. These results propose an alternative pathway implicating reduced mitochondrial energy transformation capacity as a harbinger of telomere attrition, a process exacerbated in presence of chronic stress.

## Methods

Data for this secondary analysis study were derived from a larger longitudinal study called “Stress, Aging, and Emotions (SAGE)”, which focused mainly on the effects of caregiving stress on cellular aging, and was composed by a total of 183 mothers (92 stressed caregiver mothers and 91 control mothers). All individuals were recruited in the San Francisco Bay Area, either by schools, mailing, social media, or directly through the University of California San Francisco Autism Clinic. Participants were eligible only if they were non-smokers and had an age between 20 and 50 years old, having at least one child between the ages of 2 and 16 years. For the caregiver mothers, at the beginning of the study the mean duration of years of caregiving was 5.1 years (range = 1.1–13.9). All study participants reported being premenopausal and in good health with no major medical conditions. During the time of the study, only two participants, both in the stressed caregivers group, met the diagnostic criteria for depression, and no participants were taking any hormonal birth control medication. This study was approved by the Institutional Review Board at the University of California, San Francisco, and all methods were performed in accordance with the relevant guidelines and regulations. Finally, written informed consent was obtained for each study participant.

For a subgroup of 85 participants (caregivers = 45, control = 40) we had enough preserved PBMCs to quantify the mitochondrial health index (MHI), along with the telomere length (*n* = 81) and telomerase activity (*n* = 85; see below for details). Blood samples were collected at baseline, and again 9 months later to measure changes in both telomerase activity (*n* = 75) and telomere length (*n*= 75). We were unable to measure changes in MHI at the second sampling point. There was no intervention between the time points. Both groups in this subsample did not differ in any sociodemographic or health factors [see^39^ for full details].

### Mitochondrial Health Index (MHI)

To obtain an index of mitochondrial health (MHI), we followed a protocol previously reported^39^. Briefly, to calculate the MHI we calculated 4 different parameters related to both mitochondrial function and content. As markers of mitochondrial function, we quantified the activity of two enzymes related to two complexes of the mitochondrial respiratory chain: succinate dehydrogenase (SDH) a marker of complex II activity, and cytochrome c oxidase (COX) an activity marker of complex IV. And as markers of mitochondrial content, we quantified the enzymatic activity of citrate synthase (CS) and the number of mitochondrial DNA copy number per cell (mtDNAcn). All enzymatic activities were quantified spectrophotometrically, and mtDNAcn was calculated through quantitative real-time PCR [see^39^ for details]. All four parameters were mean-centered. Then, SDH and COX were added and included as a numerator, and CS and mtDNAcn were also added and included as a denominator. As a result, our measure of MHI reflects respiratory chain capacity per unit of mitochondrial content^39^.

Our estimate of MHI was found to be affected by the time the samples spent in the freezer. However, time spent at the freezer also differed between both chronic stress groups. Thus, chronic stress status and freezer time are confounded variables so it was impossible for us to covary for freezer time in the statistical analyses without completely removing the chronic stress effect^39^.

### Telomere length and telomerase activity

Telomere length was quantified in peripheral blood mononuclear cells (PBMCs). PBMCs were isolated from whole blood samples by Ficoll Hypaque density gradient centrifugation within 6 h from blood collection, and then were conserved at -80 °C until laboratory analyses. DNA was extracted from PBMCs and then purified using the QIAamp^®^ DNA Mini Kit (QIAGEN, Hilden, Germany, Cat. Number 51104). To estimate telomere length, we used quantitative real time PCR, following a protocol previously reported^67^, which was adapted from the original method published by Cawthon^72^, which ultimately gives us a measure of telomere length controlling for a single-copy nuclear gene (T/S ratio). Eight samples were included in each plate to control for interassay variability. Each sample was measured in duplicates, and if intraassay variability was higher than 7%, a third measure was performed.

Due to logistical reasons, we analyzed all samples in two different batches separated in time. To control for variance between batches, we used the same 8 samples used to measure interassay variability in both batches. We then compared the T/S ratio of these samples from both batches, and calculated a correction factor used only for the samples in the second batch, in order to make them comparable to the samples from the first batch. The adjusted value was calculated as follows: (Second batch value – 0.04472) / 0.8676.

**Fig. 5:**
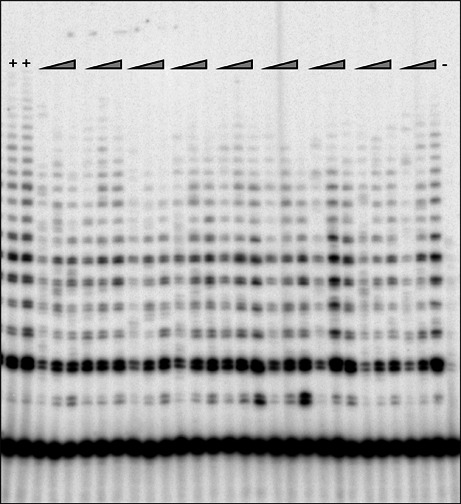
Example of a gel used for the telomerase activity estimation. In this figure, + represents the positive control, where 10 cells of 293T cancer cell line were used, - represents the negative control, and each triangle represents the increasing concentrations in the number of cells used for each focal individual (2500, 5000 and 10000 cells).

Telomerase activity was also quantified in PBMCs. Telomerase activity in PBMCs was quantified using a commercial kit (TRAPeze Telomerase Detection Kit, Millipore), where gel-TRAP assays were performed by the Telomerase Repeat Amplification Protocol (TRAP), as previously reported^67^. Briefly, after purification of the PBMCs, 5 × 10^5^–1 × 10^6^ cells per sample were pelleted and lysed with 1XCHAPS buffer. Extracts corresponding to 5000 cells/𝜇L were analyzed in batches, on an 8% polyacrylamide-8 M urea sequencing gel^67^. Then, the gel was exposed to a phosphorimager plate overnight and scanned on a Typhoon 8600 Imager (GEHealthcare, Piscataway, NJ), including both positive and negative controls along with the focal samples (Fig. 5). As a standard and positive control of telomerase activity we used the 293T cancer cell line, where estimates were expressed as equivalent of the number of 293T cells^67^. Finally, telomerase activity was quantified using the software ImageQuant 5.2 (GE Healthcare, Piscataway, NJ), where, after subtracting the background, signals from the product ladders on the gels were normalized and added against the signal from the internal control band for the same lane to get the product/internal control value, doing the same also for the negative control lane^67^.

For each telomerase activity assay reaction, the product/internal value for the sample was subtracted by the product/internal value for negative control and divided by the product/internal control value - negative control product/internal value from ten 293T cells and then multiplied by 10 to obtain the final telomerase activity units, defined as 1 unit = the amount of product from one 293T cell/10,000 immune cells^67^.

Telomerase activity and telomere length (T/S ratio) were both natural log-transformed in order to meet linear models’ assumptions.

### Statistical analytic plan

First, we tested if our two groups of chronic stress status (caregivers and controls) differed in two demographic variables: age and BMI, and in the different biological indices at baseline: MHI, telomerase activity and telomere length. This was done by doing independent t tests.

Then, we tested if our different biological indices at baseline were affected by the individuals’ age or BMI, by doing independent linear regression models where the biological indices were the response variables, and age and BMI were the explanatory terms. Later, we quantified the change in telomere length and telomerase activity over the 9-month period through the difference between both sampling points (Second measure – First measure), where negative values would indicate a decrease in either telomerase activity or telomere length, and in turn, positive values would indicate an increase. We also tested if the change in telomerase activity and telomere length were affected by the individuals’ age or BMI following the same procedure described above.

We then also explored for relationships among our biological indices at baseline. First, we tested if telomerase activity was related to MHI, and if telomere length was related to MHI and telomerase activity, by doing independent linear regression models, where chronic stress status and individuals’ age were included as covariates.

Finally, to test the direct and indirect effects that chronic stress status and MHI can have over the changes in telomerase activity and telomere length over time, as predicted in the model in Fig. 1, we performed a path analysis using the *lavaan*package version 0.6–17 in R^73^. Age was only associated with telomere length at baseline. Since age and BMI were not associated with MHI, nor with the change in both telomerase activity and telomere length over time (Table 2), they were excluded from the path analysis described below. The path analysis consisted of 3 different linear regression models testing direct effects.

The first model included the change in telomere length as the response variable, and MHI, chronic stress status and change in telomerase activity as the explanatory variables. The second model included the change in telomerase activity as the response variable, and MHI and chronic stress status as the independent terms. Finally, the third model included MHI as the response variable, and chronic stress status as the explanatory term. For exploratory purposes, we tested if the effects of MHI on the change in telomerase activity, and the effects of the change in telomerase activity on telomere length change, were dependent on the chronic stress status, by testing an interaction between these terms. However, since any of the interactions resulted significant (see Results), they were dropped from final models to remain with interpretable estimates of main effects^74^. Lastly, by using the default “Delta method” option^73^, we calculated indirect effects of: chronic stress status over changes in telomere length (through changes in telomerase activity), and MHI over changes in telomere length (through changes in telomerase activity).

In all models, we confirmed that model assumptions were met by graphical visualization of the residuals. Data were analyzed using R software version 4.1.2 (R Core Team 2021). Mean and standard deviations (SD) are shown throughout the text, and effect sizes (i.e. r^2^) of significant models are shown in the figure legends. Statistical significance was set at a p value ≤ 0.05.

## Data Availability

The datasets generated during and/or analyzed during the current study are available from the corresponding author on reasonable request.

## References

[1] Ogrodnik, M. Cellular aging beyond cellular senescence: markers of senescence prior to cell cycle arrest in vitro and in vivo. *Aging Cell.* **20**, e13338 (2021). 33711211 10.1111/acel.13338 PMC8045927

[2] Blackburn, E. H. Telomeres and telomerase: their mechanisms of action and the effects of altering their functions. *FEBS Lett.* **579**, 859–862 (2005). 15680963 10.1016/j.febslet.2004.11.036

[3] Blackburn, E. H. Structure and functions of telomeres. *Nature*. **350**, 569–573 (1991). 1708110 10.1038/350569a0

[4] Blackburn, E. H. Telomere states and cell fates. *Nature*. **408**, 53–56 (2000). 11081503 10.1038/35040500

[5] Martínez, P. & Blasco, M. A. Replicating through telomeres: a means to an end. *Trends Biochem. Sci.* **40**, 504–515 (2015). 26188776 10.1016/j.tibs.2015.06.003

[6] Campisi, J., Kim, S., Lim, C. S. & Rubio, M. Cellular senescence, cancer and aging: the telomere connection. *Exp. Gerontol.* **36** (10), 1619–1637 (2001). 11672984 10.1016/s0531-5565(01)00160-7

[7] Blackburn, E. H., Epel, E. S. & Lin, J. Human telomere biology: a contributory and interactive factor in aging, disease risks, and protection. *Science*. **350** (6265), 1193–1198 (2015). 26785477 10.1126/science.aab3389

[8] Protsenko, E., Rehkopf, D., Prather, A. A., Epel, E. & Lin, J. Are long telomeres better than short? Relative contributions of genetically predicted telomere length to neoplastic and non-neoplastic disease risk and population health burden. *PLoS ONE*. **15** (10), e0240185 (2020). 33031470 10.1371/journal.pone.0240185 PMC7544094

[9] Mons, U. et al. Leukocyte telomere length and all-cause, cardiovascular disease, and cancer mortality: results from individual-participant-data meta-analysis of 2 large prospective cohort studies. *Am. J. Epidemiol.* **185** (12), 1317–1326 (2017). 28459963 10.1093/aje/kww210 PMC5860628

[10] Wang, Q., Zhan, Y., Pedersen, N. L., Fang, F. & Hägg, S. Telomere length and all-cause mortality: a meta-analysis. *Ageing Res. Rev.* **48**, 11–20 (2018). 30254001 10.1016/j.arr.2018.09.002

[11] Bernadotte, A., Mikhelson, V. M. & Spivak, I. M. Markers of cellular senescence. Telomere shortening as a marker of cellular senescence. *AGING*. **8** (1), 3–11 (2016). 26805432 10.18632/aging.100871 PMC4761709

[12] Lee, J. J., Lee, J. & Lee, H. Alternative paths to telomere elongation. *Semin. Cell Dev. Biol.* **113**, 88–96 (2021). 33293233 10.1016/j.semcdb.2020.11.003

[13] Chan, S. R. W. L. & Blackburn, E. H. Telomeres and telomerase. *Phil Trans. Soc. Lond. B*. **359**, 109–122 (2004). 10.1098/rstb.2003.1370 PMC1693310 15065663

[14] Imamura, S. et al. A non-canonical function of zebrafish telomerase reverse transcriptase is required for developmental hematopoiesis. *PLoS ONE*. **3** (10), e3364 (2008). 18846223 10.1371/journal.pone.0003364 PMC2561060

[15] Gazzaniga, F. S. & Blackburn, E. H. An antiapoptotic role for telomerase RNA in human immune cells independent of telomere integrity or telomerase enzymatic activity. *Blood*. **124** (25), 3675–3684 (2014). 25320237 10.1182/blood-2014-06-582254 PMC4263978

[16] Thompson, C. A. H. & Wong, J. M. Y. Non-canonical functions of telomerase reverse transcriptase: emerging roles and biological relevance. *Curr. Top. Med. Chem.* **20** (6), 498–507 (2020). 32003692 10.2174/1568026620666200131125110

[17] Lin, Y. et al. Age-associated telomere attrition of lymphocytes *in vivo* is coordinated with changes in telomerase activity, composition of lymphocyte subsets and health conditions. *Clin. Sci. (Lond)*. **128** (6), 367–377 (2015). 25317735 10.1042/CS20140481 PMC5421624

[18] Lin, J., Epel, E. & Blackburn, E. Telomeres and lifestyle factors: roles in cellular aging. *Mutat. Res.* **730**, 85–80 (2012). 21878343 10.1016/j.mrfmmm.2011.08.003

[19] Lofti, R. A., El Zawahry, K. M., Kamar, Z. A. & Hashem, Z. Effects of smoking on human telomerase reverse transcriptase expression in the skin. *Int. J. Dermatol.* **53** (10), 1205–1212 (2014). 24601896 10.1111/ijd.12467

[20] Marcon, F. et al. Telomerase activity, telomere length and *hTERT* DNA methylation in peripheral blood mononuclear cells from monozygotic twins with discordant smoking habits. *Environ. Mol. Mutagen.* **58** (8), 551–559 (2017). 28843010 10.1002/em.22127

[21] Epel, E. S. et al. Accelerated telomere shortening in response to life stress. *PNAS*. **101** (49), 17312–17315 (2004). 15574496 10.1073/pnas.0407162101 PMC534658

[22] Deng, W., Cheung, S. T., Tsao, S. W., Wang, X. M. & Tiwari, A. F. Y. Telomerase activity and its associations with psychological stress, mental disorders, lifestyle factors and interventions: a systematic review. *Psychoneuroendocrinology*. **64**, 150–163 (2016). 26677763 10.1016/j.psyneuen.2015.11.017

[23] Brydon, L. et al. Hostility and cellular aging in men from the Whitehall II cohort. *Biol. Psychiatry*. **71** (9), 767–773 (2012). 21974787 10.1016/j.biopsych.2011.08.020 PMC3657139

[24] Wolkowitz, O. M. et al. Resting leukocyte telomerase activity is elevated in major depression and predicts treatment response. *Mol. Psychiatry*. **17**, 164–172 (2012). 21242992 10.1038/mp.2010.133 PMC3130817

[25] Picard, M. et al. Mitochondrial functions modulate neuroendocrine, metabolic, inflammatory, and transcriptional responses to acute psychological stress. *PNAS*. **112** (48), E6614–E6623 (2015). 26627253 10.1073/pnas.1515733112 PMC4672794

[26] Sahin, E. et al. Telomere dysfunction induces metabolic and mitochondrial compromise. *Nature*. **479**, 359–365 (2011). 21307849 10.1038/nature09787 PMC3741661

[27] Zheng, Q., Huang, J. & Wang, G. Mitochondria, telomeres and telomerase subunits. *Front. Cell. Dev. Biol.* **7**, 274 (2019). 31781563 10.3389/fcell.2019.00274 PMC6851022

[28] Vaurs, M., Dolu, E. B. & Decottignies, A. Mitochondria and telomeres: hand in glove. *Biogerontology*. **25**, 289–300 (2024). 37864609 10.1007/s10522-023-10074-7

[29] Sahin, E. & DePinho, R. A. Linking functional decline of telomeres, mitochondria and stem cells during ageing. *Nature*. **464**, 520–528 (2010). 20336134 10.1038/nature08982 PMC3733214

[30] Moslehi, J., DePinho, R. A. & Sahin, E. Telomeres and mitochondria in the aging heart. *Circul. Res.* **110** (9), 1226–1237 (2012). 10.1161/CIRCRESAHA.111.246868 PMC3718635 22539756

[31] Ahmed, W. & Lingner, J. Impact of oxidative stress on telomere biology. *Differentiation*. **99**, 21–27 (2018). 29274896 10.1016/j.diff.2017.12.002

[32] Ahmed, S. et al. Telomerase does not counteract telomere shortening but protects mitochondrial function under oxidative stress. *J. Cell. Sci.* **121** (7), 1046–1053 (2008). 18334557 10.1242/jcs.019372

[33] Haendeler, J. et al. Mitochondrial telomerase reverse transcriptase binds to and protects mitochondrial DNA and function from damage. *Arterioscler. Thromb. Vasc. Biol.* **29** (6), 929–935 (2009). 19265030 10.1161/ATVBAHA.109.185546

[34] Cheng, Y. et al. Mitochondrial trafficking and processing of telomerase RNA *TERC*. *Cell. Rep.* **24**, 2589–2595 (2018). 30184494 10.1016/j.celrep.2018.08.003

[35] Zheng, Q. et al. Mitochondrion-processed *TERC* regulates senescence without affecting telomerase activities. *Protein Cell.* **10** (9), 631–648 (2019). 30788732 10.1007/s13238-019-0612-5 PMC6711880

[36] Bobba-Alves, N. et al. Cellular allostatic load is linked to increased energy expenditure and accelerated biological aging. *Psychoneuroendocrinology*. **155**, 106322 (2023). 37423094 10.1016/j.psyneuen.2023.106322 PMC10528419

[37] Bobba-Alves, N., Juster, R. P. & Picard, M. The energetic costs of allostasis and allostatic load. *Psychoneuroendocrinology*. **146**, 105951 (2022). 36302295 10.1016/j.psyneuen.2022.105951 PMC10082134

[38] Picard, M., McEwen, B. S., Epel, E. S. & Sandi, C. An energetic view of stress: focus on mitochondria. *Front. Neuroendocr.* **49**, 72–85 (2018). 10.1016/j.yfrne.2018.01.001 PMC5964020 29339091

[39] Picard, M. et al. A mitochondrial health index sensitive to mood and caregiving stress. *Biol. Psychiatry*. **84**, 9–17 (2018). 29525040 10.1016/j.biopsych.2018.01.012 PMC6014908

[40] Trumpff, C. et al. Psychosocial experiences are associated with human brain mitochondrial biology. *PNAS*. **121** (27), e2317673121 (2024). 38889126 10.1073/pnas.2317673121 PMC11228499

[41] Gumpp, A. M. et al. Investigating mitochondrial bioenergetics in peripheral blood mononuclear cells of women with childhood maltreatment from post-parturition period to one-year follow-up. *Psychol. Med.* **53** (9), 3793–3804 (2023). 35311632 10.1017/S0033291722000411 PMC10317795

[42] Picard, M. & McEwen, B. S. Psychological stressors and mitochondria: a systematic review. *Psychosom. Med.* **80** (2), 141–153 (2018). 29389736 10.1097/PSY.0000000000000545 PMC5901654

[43] Epel, E. S. The geroscience agenda: toxic stress, hermetic stress, and the rate of aging. *Ageing Res. Rev.* **63**, 101167 (2020). 32979553 10.1016/j.arr.2020.101167 PMC7520385

[44] Lyons, C. E., Razzoli, M. & Bartolomucci, A. The impact of life stress on hallmarks of aging and accelerated senescence: connections in sickness and in health. *Neurosci. Biobehavioral Reviews*. **153**, 105359 (2023). 10.1016/j.neubiorev.2023.105359 PMC10592082 37586578

[45] Epel, E. S. & Prather, A. A. Stress, telomeres, and psychopathology: toward a deeper understanding of a triad of early aging. *Ann. Rev. Clin. Psychol.* **14**, 371–397 (2018). 29494257 10.1146/annurev-clinpsy-032816-045054 PMC7039047

[46] Lin, J. & Epel, E. Stress and telomere shortening: insights from cellular mechanisms. *Ageing Res. Rev.* **73**, 101507 (2022). 34736994 10.1016/j.arr.2021.101507 PMC8920518

[47] Deng, W. et al. Telomerase activity and its association with psychological stress, mental disorders, lifestyle factors and interventions: a systematic review. *Psychoneuroendocrinology*. **64**, 150–163 (2016). 26677763 10.1016/j.psyneuen.2015.11.017

[48] Spivak, I. M., Mikhelson, V. M. & Spivak, D. L. Telomere length, telomerase activity, stress, and aging. *Adv. Gerontol.* **6**, 29–35 (2016). 28509478

[49] de Punder, K. et al. Stress and immunosenescence: the role of telomerase. *Psychoneuroendocrinology*. **101**, 87–100 (2019). 30445409 10.1016/j.psyneuen.2018.10.019 PMC6458519

[50] Epel, E. S. et al. Dynamics of telomerase activity in response to acute psychological stress. *Behav. Immun.* **24** (4), 531–539 (2010). 10.1016/j.bbi.2009.11.018 PMC2856774 20018236

[51] Damjanovic, A. K. et al. Accelerated telomere erosion is associated with a declining immune function of caregivers of Alzheimer’s disease patients. *J. Immunol.* **179** (6), 4249–4254 (2007). 17785865 10.4049/jimmunol.179.6.4249 PMC2262924

[52] Epel, E. S. et al. Cell aging in relation to stress arousal and cardiovascular disease risk factors. *Psychoneuroendocrinology*. **31** (3), 277–287 (2006). 16298085 10.1016/j.psyneuen.2005.08.011

[53] Zalli, A. et al. Shorter telomeres with high telomerase activity are associated with raised allostatic load and impoverished psychosocial resources. *PNAS*. **111** (12), 4519–4524 (2014). 24616496 10.1073/pnas.1322145111 PMC3970484

[54] Simon, N. M. et al. Telomere length and telomerase in a well-characterized sample of individuals with major depressive disorder compared to controls. *Psychoneuroendocrinology*. **58**, 9–22 (2015). 25932992 10.1016/j.psyneuen.2015.04.004 PMC4461511

[55] Walia, N. et al. Telomerase enzyme activity in patients with major depressive disorder: A pre and post-treatment study. *J. Affect. Disord.* **320**, 268–274 (2023). 36191646 10.1016/j.jad.2022.09.138

[56] Radin, R. M. et al. Maternal caregivers have confluence of altered cortisol, high reward-driven eating, and worse metabolic health. *PLOS ONE* **14** (8), e0221354 (2019). 31412085 10.1371/journal.pone.0221354 PMC6693881

[57] Knezevic, E. et al. The role of cortisol in chronic stress, neurodegenerative disease, and psychological disorders. *Cells*. **12** (23), 2726 (2023). 38067154 10.3390/cells12232726 PMC10706127

[58] Aschbacher, K. et al. Chronic stress increases vulnerability to diet-related abdominal fat, oxidative stress, and metabolic risk. *Psychoneuroendocrinology*. **46**, 14–22 (2014). 24882154 10.1016/j.psyneuen.2014.04.003 PMC4104274

[59] Kurz, D. J. et al. Chronic oxidative stress compromises telomere integrity and accelerates the onset of senescence in human endothelial cells. *J. Cell Sci.* **117** (11), 2417–2426 (2004). 15126641 10.1242/jcs.01097

[60] Choi, J., Fauce, S. R. & Effros, R. B. Reduced telomerase activity in human T lymphocytes exposed to cortisol. *Brain. Behav. Immun.* **22** (4), 600–605 (2008). 18222063 10.1016/j.bbi.2007.12.004 PMC2386249

[61] Noguera, J. C., da Silva, A. & Velando, A. Egg corticosterone can stimulate telomerase activity and promote longer telomeres during embryo development. *Mol. Ecol.* **31** (23), 6252–6260 (2022). 33065771 10.1111/mec.15694

[62] Tyrka, A. R. et al. Alterations of mitochondrial DNA copy number and telomere length with early adversity and psychopathology. *Biol. Psychiatry*. **79** (2), 78–86 (2016). 25749099 10.1016/j.biopsych.2014.12.025 PMC4503518

[63] Conklin, Q. A. et al. Meditation, stress processes, and telomere biology. *Curr. Opin. Psychol.* **28**, 92–101 (2019). 30553080 10.1016/j.copsyc.2018.11.009 PMC6526075

[64] Sturm, G. et al. A multi-omics longitudinal aging dataset in primary human fibroblasts with mitochondrial perturbations. *Sci. Data*. **9**, 751 (2022). 36463290 10.1038/s41597-022-01852-y PMC9719499

[65] Sturm, G. et al. OxPhos defects cause hypermetabolism and reduce lifespan in cells and in patients with mitochondrial diseases. *Commun. Biology*. **6**, 22 (2023). 10.1038/s42003-022-04303-x PMC9837150 36635485

[66] Weng, N. Telomere and adaptive immunity. *Mech. Ageing Dev.* **129**, 60–66 (2008). 18199471 10.1016/j.mad.2007.11.005 PMC2276146

[67] Lin, J. et al. Analyses and comparisons of telomerase activity and telomere length in human T and B cells: insights for epidemiology of telomere maintenance. *J. Immunol. Methods*. **352**, 71–80 (2010). 19837074 10.1016/j.jim.2009.09.012 PMC3280689

[68] Faas, M. M. & de Vos, P. Mitochondrial function in immune cells in health and disease.. *Biochimica et Biophysica Acta (BBA) – Mol Basis Dis.* **1866**(10), 165845 (2020). 10.1016/j.bbadis.2020.165845 32473386

[69] Liu, C. C. et al. Immune mitochondrial phenotypes are largely preserved in mitochondrial diseases and do not reflect disease severity. Manuscript in progress (2024). 10.1212/NXG.0000000000200343 PMC12758130 41488384

[70] Rausser, S. et al. Mitochondrial phenotypes in purified human immune cell subtypes and cell mixtures. *Elife*. **10**, e70899 (2021). 34698636 10.7554/eLife.70899 PMC8612706

[71] Saretzki, G. & Wan, T. Telomerase in brain: the new kid on the block and its role in neurodegenerative diseases. *Biomedicines*. **9** (5), 490 (2021). 33946850 10.3390/biomedicines9050490 PMC8145691

[72] Cawthon, R. M. Telomere measurement by quantitative PCR. *Nucleic Acids Res.* **30**, e47 (2002). 12000852 10.1093/nar/30.10.e47 PMC115301

[73] Rosseel, Y. Lavaan: an R package for structural equation modeling. *J. Stat. Softw.* **48** (2), 1–36 (2012).

[74] Engqvist, L. The mistreatment of covariate interaction terms in linear model analyses of behavioral and evolutionary ecology studies. *Anim. Behav.* **70**, 967–971 (2005).

